# Roles of Exosome Genomic DNA in Colorectal Cancer

**DOI:** 10.3389/fphar.2022.923232

**Published:** 2022-06-01

**Authors:** Xiaoshuai Li, Qiushi Wang, Rui Wang

**Affiliations:** ^1^ Department of Blood Transfusion, Shengjing Hospital of China Medical University, Shenyang, China; ^2^ Department of Stem Cells and Regenerative Medicine, Key Laboratory of Cell Biology, National Health Commission of China, and Key Laboratory of Medical Cell Biology, Ministry of Education of China, China Medical University, Shenyang, China

**Keywords:** colorectal cancer, exosome, genomic DNA, liquid biopsy, tumor immunity

## Abstract

Exosomes are extracellular vesicles that mediate cell-to-cell communication. Bioactive substances such as DNA, RNA, lipids, and proteins are present in it, and they play an essential role in the pathogenesis of colorectal cancer (CRC). The role of RNA and protein in exosomes has been extensively studied. Exosome DNA has recently attracted the attention of a great deal of scientists. According to studies, exosome DNA mainly contains genomic DNA (gDNA) and mitochondrial DNA (mtDNA), of which exosome gDNA is widely used in liquid biopsy of CRC. It includes a variety of clinically relevant tumor-specific mutation genes. In addition to liquid biopsy, researchers find that exosome gDNA regulates immune and metabolic functions in CRC, making it an important research object. However, the primary research on exosome gDNA is still limited. Here, we describe the occurrence and composition of exosomes. Summarize the essential characteristics and mode of action of exosome gDNA. Remarkably, this paper constitutes a comprehensive summary on the role of exosome gDNA on CRC with the intent of providing a theoretical basis and reference for early diagnosis and clinical treatment of cancer.

## Introduction

CRC is the third most common malignant tumor in the world. Every year one million people worldwide will develop CRC (Franke et al., 2019; Keum and Giovannucci, 2019). It is a heterogeneous intestinal epithelial disease characterized by the accumulation of mutations and disordered immune responses (Janney et al., 2020; Lichtenstern et al., 2020; Stoffel and Murphy, 2020). Currently, the choice of treatment scheme for CRC is mostly determined by tissue biopsy. Despite tissue biopsy being the gold standard for diagnosis, classification, and treatment decision-making, it is not always available. Tumor tissue also exhibits solid spatial heterogeneity due to the uneven distribution of tumor subclones. Tumor molecular compositions may change dynamically in response to micro-environmental stimuli and therapeutic pressure, making tissue biopsy an unreliable method of diagnosis. Therefore, liquid biopsy is becoming increasingly popular (Dekker and Rex, 2018). A liquid biopsy is a non-invasive procedure in which samples of blood or other body fluids are collected to analyze exosomes, circulating tumor cells, and ctDNA (Marcuello et al., 2019). In the early stages of CRC, there are no obvious symptoms. Most patients with CRC are found in the advanced stage. Liquid biopsy provides a more accurate picture of tumor details in real time, which is particularly important for early detection of cancer and reduction of mortality from it (Marcuello et al., 2019; Kolencik et al., 2020; Martini et al., 2020; Rodriguez-Casanova et al., 2021). Nevertheless, the key to prolonging the survival time of CRC patients is not only to make early diagnosis of the disease, but also to understand the tumor progression (Ogunwobi et al., 2020; Sveen et al., 2020). The identification of a sensitive biomarker is important for observing the immune response and metabolism during the treatment of CRC and assessing the drug resistance and heterogeneity further (Marcuello et al., 2019; Kolencik et al., 2020; Martini et al., 2020; Rodriguez-Casanova et al., 2021).

Exosomes are extracellular nano-sized vesicles containing DNA, RNA, lipids, and protein (Pegtel and Gould, 2019; Jafari et al., 2020; Kalluri and Lebleu, 2020; Ahmadi and Rezaie, 2021; Liu et al., 2021; Yu et al., 2021; Rezaie et al., 2022; Vahabi et al., 2022). CRC exosomes have the ability to transport their contents to recipient cells in the tumor microenvironment, thereby playing an important role in cell-to-cell communication. It plays a vital role in tumor immunity, tumor survival, tumor chemotherapy resistance, and metastasis (Xiao et al., 2020; Liu et al., 2021; Chang et al., 2021). In CRC exosomes, there are a variety of active substances. DNA is one of the most stable substances (Jeppesen et al., 2019). However, there is little research on exosome DNA compared to proteins and RNA. Essentially, exosome DNA is derived from normal DNA metabolism or damage induction, primarily from gDNA from the nucleus and mtDNA from the mitochondria (Kahlert et al., 2014; Thakur et al., 2014; Kalluri and Lebleu, 2016). The gDNA of exosomes contains DNA fragments from multiple chromosomes, including mutant DNA fragments, and is mainly found in body fluids or the genome of some immune cells (Thakur et al., 2014; Waldenstrom et al., 2012) (Table 1). gDNA from exosomes is widely used in liquid biopsy, it also affects tumor immunity and metabolism (Lian et al., 2017; Vanpouille-Box et al., 2017; Zhang et al., 2018; Sharma and Johnson, 2020; Zhao et al., 2021). In this review, we discuss the relevance of exosome gDNA in the early detection, development and prognosis, immune response, and drug resistance of CRC.

**TABLE 1:** Exosome gDNA as biomarkers for diagnosis of diseases.

Disease	Mutantion	Detection mode	Biofluid	Reference
Pancreatic Cancer	KRAS TP53	ddPCR	Plasma	(Kahlert et al., 2014; Yang et al., 2017)
Glioma	EGFR	Conventional PCR	Cerebrospinal fluid	Vaidya and Sugaya, (2020)
non-small cell lung cancer	EGFR	PNA-PCR	Alveolar lavage fluid and Plasma	Hur et al. (2018)
Lung Adenocarcinoma	EGFR	PNA-PCR	Pleural effusion	Lee et al. (2018)
Pancreatic cancer	KRAS	ddPCR	Plasma	Allenson et al. (2017)
Pulmonary adenocarcinoma	EGFR	ddPCR	Bronchial Washing	Park et al. (2020)
Glioma	RET HIF2A VHL SDHB	Conventional PCR	Plasma	Wang et al. (2018)
non-small cell lung cancer	EGFR	ddPCR	Plasma	Castellanos-Rizaldos et al. (2018)
non-small cell lung cancer	EGFR	ddPCR	plasma and pleural fluid	Kim et al. (2021)
Lung Adenocarcinoma	EGFR	Quantitative PCR	Pleural Effusions	Qu et al. (2019)
Neuroblastoma	BRAF	Quantitative PCR	Plasma	Degli et al. (2021)
Bladder cancer	KRAS	ddPCR	Urine	Zhou et al. (2021)
prostate cancer	PTEN TP53	Quantitative PCR	Plasma	Lazaro-Ibanez et al. (2014)
Colorectal cancer	KRAS	ddPCR	Plasma	Lucchetti et al. (2021)
non-small cell lung cancer	EGFR	PNA-PCR	bronchoalveolar lavage fluid	Hur et al. (2019)
non-small cell lung cancer	EGFR	ddPCR	Plasma	Krug et al. (2018)
Pancreatic cancer	KRAS	ddPCR	Plasma	Bernard et al. (2019)
Glioma	IDH1	ddPCR	Plasma	Garcia-Romero et al. (2017)

## Overview of Exosomes

Exosomes are extracellular nano-sized bilayer membrane vesicles with an average diameter of 30–150 nm (Pegtel and Gould, 2019; Kalluri and Lebleu, 2020; Yu et al., 2021). It is produced in the cytoplasm by the classical endosomal sorting complex. It is formed by the inward budding of cell membranes containing ubiquitinated surface receptors, leading to the formation of early endosomes. With the help of the Golgi apparatus, these early endocorpuscles become late endocorpuscles and intracavitary vesicles. Intracavitary vesicles accumulate in endosomes, leading to the formation of multivesicular bodies (MVB). MVB is ultimately transported into lysosomes for degradation or fuses with the cytoplasmic membrane, releasing its contents (including exosomes) into the extracellular space (He et al., 2018; Doyle and Wang, 2019; Zhang and Yu, 2019; Rezaie et al., 2021) (Figure 2). Exosomes are identified by classical molecular markers, such as tetrapeptide (CD63, CD9, CD81), FLOTILIN-1, and heat shock 70 protein (Liang et al., 2021). Numerous literature has been extensively studied to reveal the detailed mechanism of exosome biogenesis.

Exosomes can be found in almost all cells and body fluids, including blood, sweat, tears, urine, saliva, breast milk, ascites, and cerebrospinal fluid (Liang et al., 2021; Lin et al., 2021). Exosomes carry various macromolecules from different tissues and organs, loaded with nucleic acids (DNA and RNA), structural components of cells (protein and lipids), and cell metabolites. It is a part of the intercellular communication system, which carries and transmits signal molecules that regulate the physiological state of cells, participates in antigen presentation, cell differentiation, growth, and tumor immunity, and is closely related to the occurrence and development of various diseases (Pegtel and Gould, 2019; Liu et al., 2021). After different active substances in exosomes are thrown out of cells, functional protein, RNA, and DNA fragments can be transferred to recipient cells, resulting in cascade changes in the genome and non-genome (Liu et al., 2021; Kalluri and Lebleu, 2020) (Table 2). Various bioactive molecular substances in exosomes are cell signaling effectors and valuable tools for tumor diagnosis. Exosomes can monitor the occurrence and development of tumors and provide new targets and strategies for tumor treatment and diagnosis (Liang et al., 2021; Lin et al., 2021).

**TABLE 2:** Exosome gDNA transfer between cells.

Donor cell	Receptor cell	Exosome gDNA	Integrated into the genome	Reference
Cardiomyocyte	Fibroblast	Non specicial	Not sure	Waldenstrom et al. (2012)
Colon cancer cell	Fibroblast and epithelial cells	HRAS	No	Lee et al. (2016)
Glioma cells	Fibroblast	c-Myc	Yes	Balaj et al. (2011)
K562	HEK293	AT1	Yes	Cai et al. (2013)
SW480	*In vivo*	KRAS RAB	Not sure	Trejo-Becerril et al. (2012)
hBMSC	hMSC	A.t.-plasmid	Yes	Fischer et al. (2016)
H-ras-driven Intestinal epithelial cells	RAT-1	HRAS	No	Lee et al. (2014)
K562	Neutrophils	BCR/ABL	Yes	Cai et al. (2014)

## Origin and Characteristics of Exosome Genomic DNA

Exosomes contain many bioactive substances. However, compared with proteins and RNA in exosomes, there is little research on exosome DNA. Essentially, mammalian cells can excrete harmful cytoplasmic DNA through exosomes, thus avoiding cell aging and death (Takahashi et al., 2017). However, tumor cells often accumulate damaged DNA fragments of the cytoplasm because of their aging or abnormal leakage of DNA during division, autophagy, oxidative stress (Riches et al., 2014; Yokoi et al., 2019). For these reasons, tumor cells can secrete more exosomes to avoid the accumulation of nuclear and mitochondrial DNA fragments in the cytoplasm induced by metabolic stress, thus releasing more soluble DNA fragments (Parolini et al., 2009; Whiteside, 2016; Yokoi et al., 2019). There are single-stranded and double-stranded DNA in tumor exosomes (Balaj et al., 2011; Cai et al., 2013; Hur and Lee, 2021), and gDNA, mtDNA, and plasmid DNA all have been found in tumor exosomes (Erdmann et al., 2017; Sansone et al., 2017; Lazaro-Ibanez et al., 2019; Elzanowska et al., 2021).

After analyzing the exosomes in the supernatants of various tumor cell lines and non-tumor-related fibroblasts, scientists found that the exosome DNA in tumor cells are more abundant (Kahlert et al., 2014; Thakur et al., 2014; Kalluri and Lebleu, 2016). Genome-wide sequencing showed that tumor exosomes contained large double-stranded gDNA fragments ranging from 100bp to 20kbp, which could cover the whole chromosome range and reflect the mutation state of tumor parent cells (Kahlert et al., 2014; Thakur et al., 2014; Kalluri and Lebleu, 2016). This gDNA is packed in exosomes in the form of nucleosomes or supercoils. As exosomes contain larger gDNA fragments and originate from living cells, which is more conducive to mutation detection by PCR, they may have advantages over circulating cfDNA (Kahlert et al., 2014; Thakur et al., 2014; Kalluri and Lebleu, 2016). Some of its tumor-related mutations can reflect the progress and prognosis of many kinds of tumors (Table 1), used for molecular map analysis of tumors. As the double-stranded gDNA is very stable. The membrane of the exosome can protect the nucleic acid substances inside it from degradation induced by nuclease. It is also reported that exosome gDNA in serum can remain stable for 1 week at 4°C and 1 day at room temperature, even after repeated freezing and thawing cycles (Jin et al., 2016), so exosome gDNA is more stable. Based on these advantages, scientists can do more in-depth research on it, whether it is screening for early diseases, monitoring drug resistance, or evaluating prognosis, which has its significance.

## Extraction, Detection, and Functional Mechanism of Exosome Genomic DNA

**FIGURE 1:**
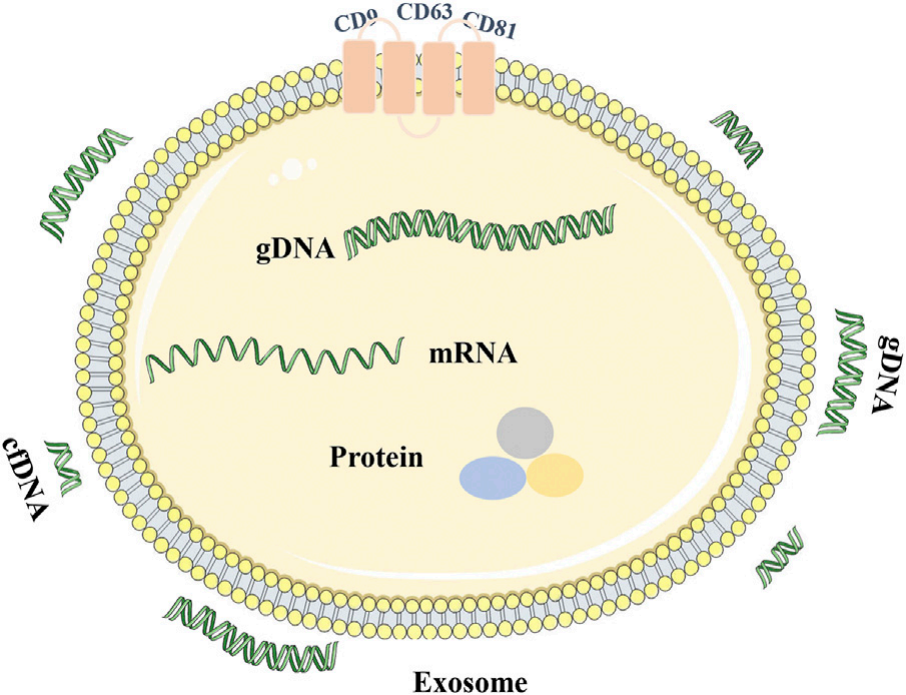
Characterization of exosome gDNA. The short double-stranded DNA is cfDNA and the long double-stranded DNA is gDNA. DNA can be enclosed within exosome, attached to the outer surface of exosome or enclosed and attached to the outer surface of exosome.

As the study of exosome gDNA has attracted extensive attention from scientists, the exosome separation and subsequent exosome gDNA detection still require more specialized studies to ensure optimal performance. When analyzing the gDNA inside exosomes, digestion outside of exosomes by DNase I should be the first procedure. DNase I can reduce the residue of cfDNA in samples and the pollution of DNA outside of exosomes (Spada et al., 2020; Wang et al., 2020) (Figure 1). It has been reported that the number of mutant DNA will be increased if DNase I is not pretreatment. Next, gDNA was extracted from exosomes which were treated with DNase I. The genomic DNA kit for tissues, cells, and blood can be used to extract gDNA from exosomes. Scientists using the Qiagen kit could get a higher concentration of exosome gDNA, but the Qiagen kit may be a bit expensive (Thakur et al., 2014; Kalluri and Lebleu, 2016; Spada et al., 2020; Wang et al., 2020). Our laboratory found that applying the inexpensive Tiangen kit can also successfully obtain a higher concentration of exosome gDNA. Typically, extracting 200 μl exosome according to the instructions will yield at least 20 ng/μl gDNA. After removing gDNA from exosomes, mutation detection of exosome gDNA is carried out. Most detection methods are digital PCR and fluorescence PCR to ensure more accurate mutation detection (Table 1). However, when the mutation to be detected is a point mutation, Sanger sequencing should be adopted as far as possible, and then T-A clones obtained a large number of clones for follow-up detection.

**FIGURE 2:**
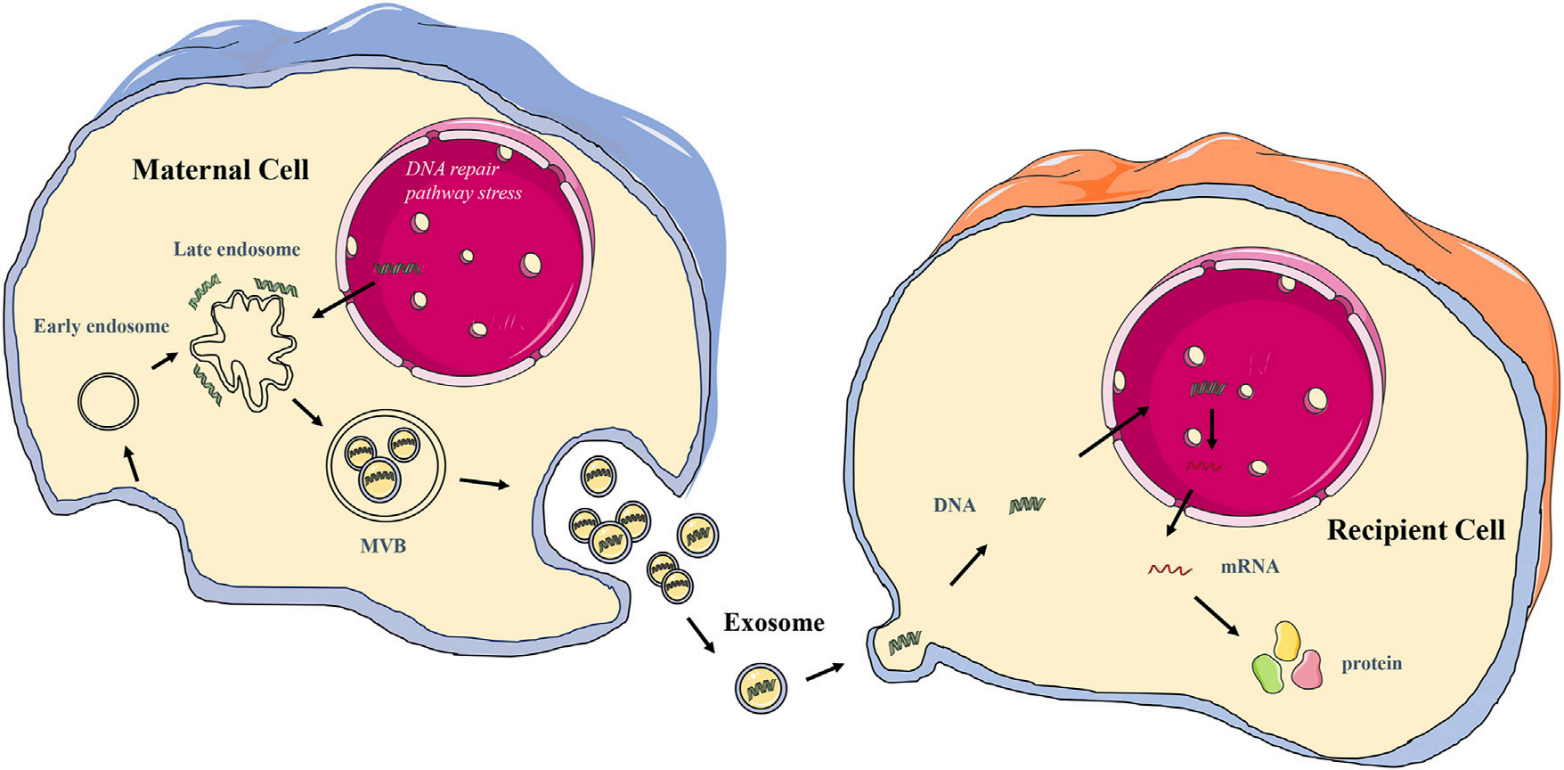
The schematic diagram of exosome gDNA transfer between cells.

Exosomes are an essential form of intercellular communication. Exosomes can deliver both RNA and protein as well as DNA (Kalluri and Lebleu, 2020) (Table 2). The transferred exosome gDNA affects the function of recipient cells by increasing mRNA transcription and protein translation (Cai et al., 2013; Cai et al., 2014; Lee et al., 2014; Fischer et al., 2016). The schematic diagram of the release of exosome gDNA is displayed in Figure 2. In addition to the open reading frame, exosomes should also contain the 5′ promoter region and 3′ untranslated region elements necessary for the transcription mechanism so that the delivered DNA can perform its function. It has been reported that there are DNA fragments in exosomes, including the 5′ promoter region, 3′ untranslated region, and active retrotransposon, which indicates that exosome gDNA may play a role in the genetic instability of receptor cells (Balaj et al., 2011; Cai et al., 2013). It has also been confirmed that exosome gDNA can be located in the recipient nucleus through late endosomal transport related to nuclear membrane invagination (Waldenstrom et al., 2012; Cai et al., 2013). According to these viewpoints, it shows that the function of oncogenes in tumor cells is not only to accumulate in the genome of tumor cells but also to transfer exosome gDNA and spread in tumor and normal cells (Table 2), which may lead to the tumorigenic transformation of normal cells and accelerate the progress of diseases. However, whether the tumor exosome gDNA can be horizontally transmitted to the recipient cells or the changes in the function of the recipient cells caused by the horizontal transmission are still controversial. In terms of the types of recipient cells, it has been reported that not all recipient cells can be horizontally transmitted. When the recipient cells are tumor cells, fibroblasts, or endothelial cells, the exosome gDNA can be easily shared, but it cannot be transmitted when the recipient cells are dormant cells such as epithelial cells (Lee et al., 2016); In the aspect of functional changes after horizontal transmission, some reports suggest that the gDNA transmitted to the recipient cells can be integrated into the genome of the recipient cells for a long time (Balaj et al., 2011; Cai et al., 2013; Cai et al., 2014; Fischer et al., 2016). Some reports suggest that the transmitted gDNA enters the recipient cells only for an instant function and loses its position after 1 month (Trejo-Becerril et al., 2012; Lee et al., 2014; Lee et al., 2016). There is also a report suggests that the transmitted gDNA is a driving factor rather than an initial factor, and only obtaining this driving factor can make the recipient cells deteriorate (Stefanius et al., 2019). The specific mechanism needs further study. From these perspectives, exosome gDNA horizontally may represent a new method of gene transfer and signal transduction between cells, which may be an essential mechanism of tumor occurrence, development, and metastasis, and provide a new direction for discovering new disease mechanisms and developing new treatment strategies.

## Colorectal Cancer Microenvironment Plays an Essential Role on Exosome Genomic DNA Packaging and Transmission

Hypoxia and acidification stimulate the tumor microenvironment, further activate the cell stress response mechanism, and increase the production of exosomes (Hoshino et al., 2013; Khaksar et al., 2018; Panigrahi et al., 2018; Soraya et al., 2021). At the same time, it may promote the package of gDNA into exosomes, and transfer it to the recipient cells, thus enhancing the ability of exosomes to induce malignant transformation of the recipient cells (Nemeth et al., 2017).

It has been reported that TP53 mutation in the recipient cells increased after the treatment factors affected the tumor microenvironment, and the transcription in colon epithelial cells and liver cells increased. The difference in mutant TP53 between colon epithelial cells and liver cells was also observed. It seems that liver cells are less likely to absorb mutant genes carried by exosomes than colon epithelial cells. The author also proved that LPS activated TLR4 of colon cancer cell SW480 and promoted the packaging of mutant TP53 in exosomes but could not promote the selective packaging of the KRAS gene. It indicates that the mechanism of gDNA packaging to exosomes is not random (Domenis et al., 2021). More and more experiments proved that exosomes derived from primary tumors could be loaded with specific molecules (Stobiecka et al., 2019; Baris et al., 2021), and the expression or lack of these molecules is helpful to the phenotype transformation of recipient cells. These results indicate that the tumor microenvironment increases the possibility of tumor gene integration by promoting exosome metastasis. Although the integration of mutant genes is still considered a rare event, it may be a related phenomenon *in vivo*. Further studies are needed to evaluate the effect of tumor-driven genes transferred through the exosome on malignant transformation and their role in tumor progression of microenvironmental stimulation.

## Exosome Genomic DNA as a Tool for Colorectal Cancer Liquid Biopsy

Due to the lack of mismatch repair function in CRC, genome instability often leads to the accumulation of frameshift mutation in the microsatellite region. And CRC in an early stage can be prevented and cured, so it is necessary to explore non-invasive tools to help with early detection and treatment monitoring (Janney et al., 2020; Stoffel and Murphy, 2020). A liquid biopsy is a dynamic tool for the non-invasive detection of tumor heterogeneity and mutation over time. Studies have shown that compared with the healthy group, the circulating exosomes in the body fluid of tumor patients are more, and exosome gDNA may provide information about tumor-specific mutations. The relationship between mutated gDNA and types of body fluids is summarized below (Table 1). Therefore, detecting exosome gDNA mutations in tumor progression can help diagnosis early-stage, drug selection, and prognosis analysis.

EGFR mutation was seen in exosomes of malignant pleural effusion (Lee et al., 2018), and T790M mutation was also detected in exosomes of bronchoalveolar lavage fluid of patients with non-small cell lung cancer (Park et al., 2020). The exosome gDNA isolated from ascites of ovarian cancer patients reflects the copy number variation of primary tumors (Crow et al., 2017; Giannopoulou et al., 2019). KRAS mutation is also confirmed in exosomes of pancreatic ductal adenocarcinoma in the early stage and the late phase (Allenson et al., 2017). The serum exosomes of glioma patients also carry the gDNA sequence with the same biological mutation as glioblastoma (Wang et al., 2018; Vaidya and Sugaya, 2020). In addition, the corresponding driving mutation was also found in the exosomes of prostate cancer (Lazaro-Ibanez et al., 2014). The genome-wide sequencing of exosomes of these tumor patients may provide information for diagnosis and prediction results.

In CRC, KRAS and BRAF mutation was detected from the serum exosomes of CRC patients with higher sensitivity and specificity (Hao et al., 2017; Lucchetti et al., 2021). In addition, it has been reported that transforming growth factor receptor 2 (TGFR2) is a part of the key signal pathway in colon epithelial cells. Its double allele frameshift mutation occurs repeatedly in most colorectal tumors, which is thought to drive colorectal cancer. It has been found that the frameshift mutation in the microsatellite region of TGFR2 is wrapped in the exosome derived from CRC cells. Although it can be detected at the DNA level, the author has not observed any frameshift protein in the exosome protein group. And then, acting on the receptor cells can up-regulate the expression of cytokines (Fricke et al., 2017). It has also been reported that the level of exosome gDNA of patients with KRAS mutation in CRC increases significantly in the course of the disease and shows more changes after treatment. Moreover, in CRC patients who meet the surgical conditions, the tumor size is related to the copy number of KRAS mutation. The copy number and abundance score of KRAS mutation in exosomes of patients with liver metastasis is significantly increased. The author also confirmed that KRAS mutation disappeared rapidly after the first chemotherapy cycle. However, it is necessary to closely monitor secondary drug resistance after anti-EGFR treatment to stop this treatment as soon as possible (Lucchetti et al., 2021). Therefore, exosome gDNA can be used as an innovative tool to monitor the diagnosis, drug resistance, and prognosis of CRC patients during treatment.

## Exosome Genomic DNA Regulates Immunity and Metabolism of Colorectal Cancer

Tumor immunosuppression is a sign of cancer progression, signaled by immune checkpoints on immune cell subsets (Nikfarjam et al., 2020). In recent years, targeted checkpoint immunotherapy has therapeutic effects on cancer patients (Li et al., 2016; Li et al., 2018). Exosomes play a significant role in tumor immunity by paracrine signal regulators. Among many components of exosomes, exosome gDNA can be used as a potent regulator of the STING pathway, which can regulate tumor immunity. During radiotherapy, gDNA fragments accumulated in the cytoplasm of tumor cells can induce an IFN-1 response and then activate dendritic cells through paracrine to trigger an anti-tumor response. This process is regulated by the feedback loop that DNA exonuclease Trex1 degrades cytosolic DNA fragments to prevent anti-tumor reactions. However, to avoid this phenomenon, tumor cells can export gDNA pieces to the exosome. Once the exosome gDNA is internalized by tumor-infiltrated dendritic cells, it will activate the STING signal path in dendritic cells. Once dendritic cells are activated by exosome gDNA, they will produce IFN-1 and recruit more CD8+T lymphocytes to prevent tumor growth further (Vanpouille-Box et al., 2017). Crohn’s disease is related to the risk of colorectal cancer. Exosome gDNA internalized into macrophages can activate the STING signal path. The research also shows that exosome gDNA can’t work after inhibiting STING, which further confirms the importance of the STING pathway in exosome gDNA. It shows that exosome gDNA plays a vital role in the immunity of CRC and can be used as a potential biomarker and therapeutic target for Crohn’s disease (Zhao et al., 2021). Reports suggest CRC will produce intestinal syndrome after being treated with irinotecan and fluorouracil, and the severity of diarrhea is closely related to the content of exosome gDNA. It is suggested that drug therapy can trigger the release of gDNA by intestinal epithelial cells through exosomes. Then the AIM2 inflammasome in immune cells will be activated, which will promote the body’s inflammatory response. Therefore, exosome gDNA is not only a direct tumor immunomodulatory but also can affect the inflammatory reaction related to chemotherapy (Lian et al., 2017). Exosomes gDNA can indeed destroy the influence of checkpoint inhibitors. Therefore, in drug development, we should pay more attention to the signal of cell autonomic paracrine. In CRC, KRAS mutation will participate in metabolism *in vitro* and *vivo* by inducing glucose transporter 1 (Zhang et al., 2018). However, it is not sure if the exosome gDNA works, which needs further study in tumor metabolism.

## Further Perspective

The discovery of high-frequency tumor mutant genes in exosome gDNA has attracted the attention of clinicians and basic researchers. Exosome gDNA is long and stable so that it can be used as excellent biomaterials for liquid biopsy (Jin et al., 2016) (Table 1). Many experts predict that in the near future, liquid biopsy based on exosome gDNA may improve the individualized treatment and prognosis of patients, especially for CRC patients, as the essential feature of CRC patients is the accumulation of mutations (Lichtenstern et al., 2020). Exosome gDNA can also be transferred from one cell to another by endocytosis or fusion. The transferred exosome gDNA can increase mRNA and protein expression in recipient cells and affect the function of recipient cells (Table 2). This may explain new pathogenesis of CRC and provide a new method for diagnosis or treatment in the future. However, it is necessary to conduct a larger-scale clinical study on these markers and targeted substances to verify the transformation of exosome gDNA into high-throughput practical solutions in the clinical environment.

Before exosome gDNA is officially applied in the clinic, it needs to meet the repeatable and verifiable conditions. However, due to the small amount of gDNA in exosomes and the different extraction methods, there is no uniform extraction and detection method, so its application is limited. Some hospitals have set up some large-scale analysis platforms to analyze it, which can provide helpful information for doctors’ diagnoses and make it possible to specialize in the analysis of exosome gDNA.

However, if we don’t know the mechanism of exosome gDNA, we can’t get the clinical effect. Therefore, the primary research on the packaging of exosome gDNA is the premise of developing the clinical treatment of exosome gDNA. Finally, further studies on the length, base composition, and stability of exosome gDNA under different physiological conditions will open up a new field for the diagnosis and treatment of colorectal cancer.

## Conclusion

**FIGURE 3:**
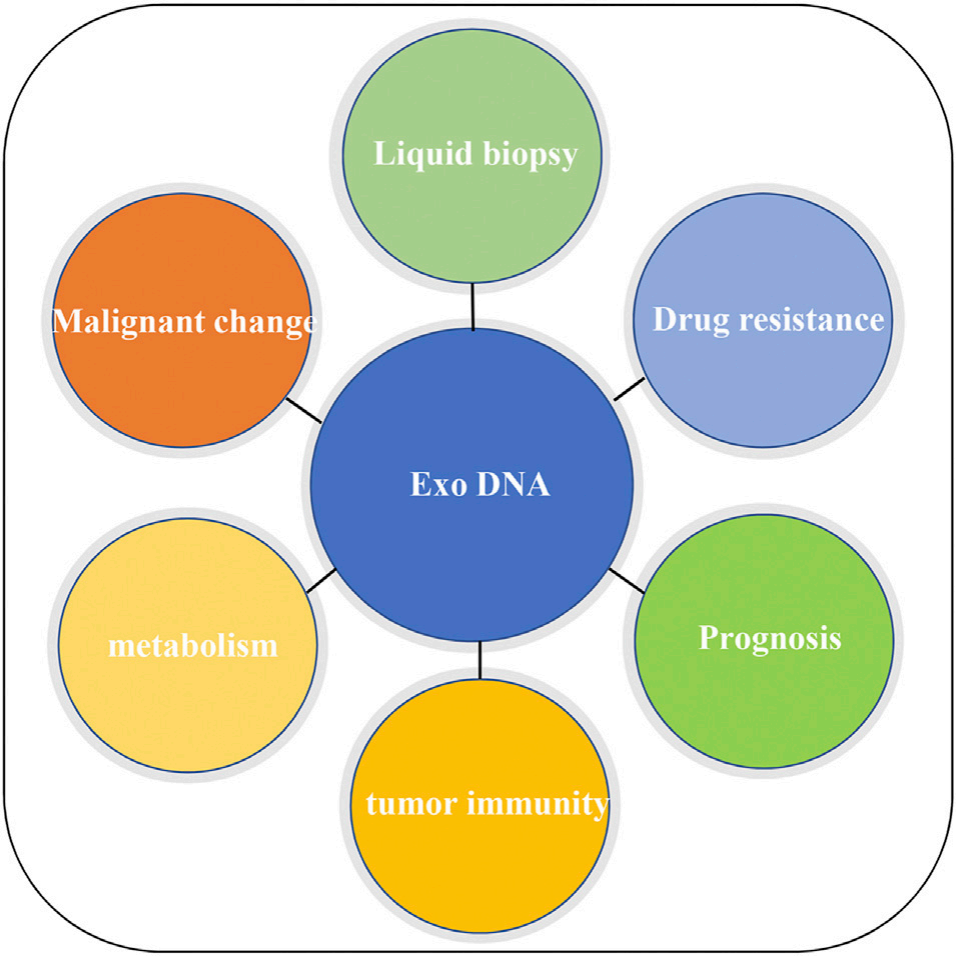
Application of exosome gDNA in CRC.

This article systematically reviewed the origin, mode of action of exosome gDNA, and its relationship with diagnosis, drug resistance, prognosis, immunity, and metabolism of CRC. The schematic diagram of the function of exosome gDNA is displayed in Figure 3. So far, we may have only seen the tip of the iceberg, and most of it is unknown. Therefore, more research is needed to reveal anonymous information about the application of exosome gDNA in the diagnosis and treatment of CRC.
